# Importance of dietary fibre, strategies for increasing intake and maintenance of the supply chain in the UK

**DOI:** 10.1098/rstb.2024.0148

**Published:** 2025-09-18

**Authors:** Julie A. Lovegrove, Kim G. Jackson, Yankho Kaimila, Stella Lignou, Alison Lovegrove, Victoria Norton, Donal M. O'Sullivan, Peter Shewry, Paola Tosi, Marcus J. Tindall

**Affiliations:** ^1^ Hugh Sinclair Unit of Human Nutrition, Department of Food and Nutritional Sciences, University of Reading, Reading RG6 6AP, UK; ^2^ Institute of Food Nutrition and Health, Department of Food and Nutritional Sciences, University of Reading, Reading RG6 6AP, UK; ^3^ Institute of Cardiovascular and Metabolic Research, University of Reading, Whiteknights, Reading RG6 6AA, UK; ^4^ Sensory Science Centre, Department of Food and Nutritional Sciences, University of Reading, Reading RG6 6AP, UK; ^5^ Rothamsted Research, Harpenden AL5 2JQ, UK; ^6^ School of Agriculture, Policy and Development, University of Reading, Whiteknights, Reading RG6 6BZ, UK; ^7^ Department of Mathematics and Statistics, University of Reading, Whiteknights, Reading RG6 6AX, UK

**Keywords:** dietary fibre, cardiovascular disease, food system, sustainable, pulses, cereals

## Abstract

Currently, dietary fibre intakes within UK populations are far below those recommended for reducing chronic disease risk. Dietary fibre is present predominantly in plant-based foods and is not digested or absorbed in the upper gastrointestinal tract, passing to the colon where it may be fermented by the gut microbiota. Types, contents and properties (notably solubility, viscosity and fermentability) of fibre vary considerably between food sources, which may result in different effects on human physiology. There is strong evidence for the benefits of dietary fibre, particularly cereal fibre and wholegrain, in reducing the risk of cardiometabolic diseases and colorectal cancer by increasing faecal mass, fermentation to short-chain fatty acids, lowering blood lipids and improving glycaemic control. There is, therefore, an urgent need to develop effective strategies to increase the intake of dietary fibre across the UK population. Here, we consider strategies comprising better nutritional education, public health messaging, more informative and effective food labelling, food reformulation, food fortification and biofortification, policy change and maintenance of the supply chain. Engagement of multiple stakeholders within the food system in this common ambition is essential for success. This requires transformation of the UK food system to ensure the sustainable availability of palatable, affordable, fibre-rich foods, ideally accompanied by individual motivation for dietary change.

This article is part of the theme issue ‘Transforming terrestrial food systems for human and planetary health’.

## 1. What is dietary fibre?

Dietary fibre is present almost exclusively in plant-based foods, with the content and composition varying widely between different crops and foods. Dietary fibre consists of components that are not digested or absorbed in the upper gastrointestinal (GI) tract (stomach and small intestine) and therefore pass into the colon, where they may be fermented by the gut microbiota. All definitions of dietary fibre include non-starch polysaccharides (NSPs) derived from plant cell walls but may differ in the extent to which other components are included. For example, the Cereals and Grains Association (https://www.cerealsgrains.org) definition of dietary fibre also includes resistant starch (RS) and fructans, details of which are as follows.

### (a) Cell wall polysaccharides and lignin

The major components of plant cell walls are polysaccharides, which can be divided into three types. Cellulose, present in all plant cell walls, consists of unbranched chains of β1,4-linked glucose units that form insoluble microfibrils. A range of other polysaccharides consisting of hexose and/or pentose sugars (collectively called ‘hemicelluloses’) and pectins (with three major types, homogalacturonan, rhamnogalacturonan-I and rhamnogalacturonan-II) may also be present. The CODEX [1] definition states that ‘when derived from a plant origin, dietary fibre may include fractions of lignin and/or other compounds associated with polysaccharides in the plant cell walls’. Lignin is a complex, amorphous phenolic polymer whose composition varies according to the plant species.

### (b) Oligosaccharides

Several fermentable oligosaccharides occur in plant foods, notably fructo-oligosaccharides (FOS, also called fructans) and gluco-oligosaccharides (GOS), raffinose (the trisaccharide galactose–glucose–fructose) and stachyose (the tetrasaccharide galactose–galactose–glucose–fructose).

### (c) Resistant starch

Starch is a mixture of two glucose polymers: amylose, which comprises single unbranched chains of up to several thousand glucose units, and amylopectin, which is highly branched and in some plants may comprise up to a million glucose units. Most starch is digested in the upper GI tract, but a proportion is not digested and defined as ‘RS’. RS includes starch and partial digestion products of starch that escape digestion in the small intestine and pass into the colon. It is classified into several types, including RS2 and RS3 [2]. RS2 is resistant to digestion in its native state and includes high amylose starches in cereal grains. RS3 is formed when starch is heated and then recrystallizes (retrogradation) and again contains a greater proportion of high amylose starches [3].

The term 'dietary fibre' therefore includes many different types of fibre, and it is possible that they may act individually, additively or synergistically to affect human health and disease risk.

## 2. Health effects of dietary fibre and potential mechanisms of action

There is strong evidence from prospective cohort studies for the benefits of dietary fibre, particularly cereal fibre and wholegrain, on cardiometabolic diseases (cardiovascular diseases (CVD), type 2 diabetes [4]) and colorectal cancer [5]. The types of dietary fibre and the specific foods with which they are associated are considered to be important in relation to the risk of mortality. In particular, fibre from wholegrains, cereals and vegetables has been associated with a reduced all-cause mortality in a meta-analysis of prospective cohort studies, with dietary fibre from nuts associated with a more marked reduction in the risk of CVD-related deaths than other food groups that were studied [6]. Furthermore, data from randomized controlled trials show that higher intakes of wheat and other cereal fibres increase faecal mass and decrease intestinal transit times, which are important for gut motility [7]. The favourable effects of oat bran and isolated β-glucans on blood lipid profiles (lower total cholesterol, low-density lipoprotein-cholesterol and triacylglycerol concentrations) and blood pressure have led to food-based health claims approved by government authorities including the European Food Safety Authority [8].

Possible mechanisms of action of dietary fibres are varied and often difficult to disentangle from the contributions of other components within foods, including those bound to the fibre (such as polyphenols, vitamins and minerals) and the effects of the gut microbiota. However, it is clear that mechanisms occur in different parts of the GI tract, where they: (i) regulate the rate of food breakdown; (ii) reduce the rate of food digestion and absorption in the upper GI tract to reduce glycaemic load; (iii) produce small-molecule metabolites (including short chain fatty acids) following fermentation by the gut microbiota, which protect the epithelial cells of the colon (colonocytes) and, after absorption, act as important regulators of human metabolism; and (iv) increase faecal bulk to reduce transit time and promote colon health.

The health effects of dietary fibres vary depending on the specific fibre type and food source (tables 1 and 2, respectively). Gill *et al*. [12] suggested that the balance of three properties—solubility, viscosity and fermentability in the colon—plays a key role in determining the behaviour of dietary fibres in the GI tract as detailed in table 1. The total amount of cell wall polysaccharides may affect the rate of food breakdown by impacting the mechanical properties and restricting access to digestive enzymes. For example, as described above, cellulose is completely insoluble, whereas most hemicelluloses exist in soluble and insoluble forms, with the proportions of soluble fibre varying within and between different plant species and tissues. Polysaccharides have high water-binding capacity and insoluble types may form insoluble gels. By contrast, soluble types affect the viscosity of the digestate, which may reduce the rate of digestion—including the release and uptake of glucose. This can have important implications for the glycaemic and insulinaemic responses to certain foods. Solubility will also affect fermentation, with soluble polysaccharides and oligosaccharides (FOS and GOS) being fermented more rapidly in the proximal colon and insoluble forms more slowly in the distal colon. The consequence of this includes a more balanced gut microbiota composition (e.g. increased *Bifidobacteria* and *Lactobacillus*) that favours host health via effects on the communication axis between the gut and major organs in the body, including the brain, liver and kidney [13]. However, some components associated with dietary fibre such as phytates in cereal brans can have an acute impact on the absorption of micronutrients, such as zinc and iron. This is important to consider in some population groups (such as those with micronutrient deficiencies), although there is evidence of adaptative mechanisms such as upregulation of micronutrient absorption in the long term [14].

**Table 1.** Examples of major dietary fibre types, structure and properties.

component	properties			structure
	solubility	viscosity	rate of fermentation	
**arabinoxylan**	varies up to 50% of total, cross-linked and highly substituted forms insoluble	highly viscous	varies with solubility and complexity, with insoluble highly substituted/cross-linked forms being more slowly fermented	(1→4)-β-linked xylose with (1→2)-α and (1→3)-α-l-arabinose substitutions. May be feruloylated, leading to cross-linking, and substituted with glucuronic acid.
**mixed-linkage β-glucan (cereals**)	varies from about 10% of total to most	highly viscous		(1→3,1→4)-β-linked d-glucose.
**mixed-linkage β-glucan (fungi**)	soluble	highly viscous		(1→3,1→6)-β-linked d-glucose.
**xyloglucan**	soluble	viscous		(1→4)-β-d-glucose with three out of four residues substituted with α-d-xylose at the O-6 position.
**mannan**	soluble	viscous		unsubstituted chains of (1→4)-β-d-mannose residues with low acetylation.
**glucomannan**	soluble	viscous		chains of (1→4)-β-d-mannose and d-glucose with some branches through (1→6)-β-linkages.
**rhamnogalacturonan-I (RGI**)	soluble	highly viscous solutions and gels		complex branched structure with backbone of repeated disaccharide galacturonic acid-rhamnose with arabinan and/or galactan and/or arabinogalactan side chains.
**rhamnogalacturonan-II (RGII**)				highly complex polymer of (1→4)-α-d-galacturonic acid (1→4)-α-d-galacturonic acid substituted with up to 12 different sugars with other modifications.
**homogalacturonan**				linear homopolymer of (1→4)-α-d-galacturonic acid that is partially methyl-esterified at the C-6 carboxyl.
**fructans (FOS**)	soluble	non-viscous	rapidly fermented	oligomers of 3−10 fructose units with (2→1)-β and/or (2→6)-β linkages. May have single glucose residue.
**raffinose**				trisaccharide: galactose–glucose–fructose.
**stachyose**				tetrasaccharide: galactose–galactose–glucose–fructose.
**resistant starch**	insoluble	non-viscous	moderate	25% amylose (linear α−1→4-d-glucose) and 75% amylopectin ((1→4)-α-d-glucose with about 5% (1→6)-α-linkages forming branches).
**cellulose**	insoluble	non-viscous	slowly	(1→4)-β-d-glucose.
**lignin**	no	no	no	cross-linked phenolic polymer.

**Table 2.** Examples of types and amounts of dietary fibre in widely consumed foods and portions^a^.

food types	major component (s)	foods	TDF g/100 g	typical portion size	TDF g per portion
**cereals**	arabinoxylan, fructans, β-glucan, cellulose, pectins	wholemeal bread	7	2 slices 80 g	5.6
		breakfast cereal (wholemeal)	11−15	1 bowl 40 g (dry)	4.4−6.0
		white bread	2.5	2 slices 80 g	2.0
		white pasta	1.2	1 bowl 75 g (uncooked)	0.9
		oats	5.5	1 bowl porridge oats, 45 g (uncooked)	2.5
		white rice	0.5	1 bowl 65 g (uncooked)	0.35
**legumes**	cellulose,xyloglucan, pectins, fructans	beans	~6.0	3 heaped tablespoons 60 g	3.6
		peas			
		lentils			
**fresh fruit**	cellulose, pectins	apple	2.4	1 medium apple 200 g	4.8
		bananas	2.6	1 banana (peeled) 180 g	4.7
		grapes	0.9	10−12 grapes 80 g	0.7
**nuts**	cellulose	almonds	11	a ‘handful’ 30 g	3.3
		peanuts	8.5	a ‘handful’ 30 g	2.6
**fungi**	β-glucan	mushrooms	~0.3	5−7 button mushrooms 80 g	0.24
**vegetables**	cellulose	broccoli	2.6	2 large spears 80 g	2.1
		spinach	2.8	one-half cup 80 g	2.2

^a^ Adapted from [9–11]; Portion size British Nutrition Foundation.

## 3. Dietary fibre recommendations and intake

The Scientific Advisory Committee on Nutrition (SACN) is the independent committee in the UK that advises the Office of Health Inequalities and Disparities (OHID) and the government on dietary recommendations for policy development. In 2015, SACN recommended that the definition of ‘dietary fibre’ should be updated to include all carbohydrates that are neither digested nor absorbed in the small intestine and have a degree of polymerization of three or more monomeric units, plus lignin. This is in contrast to the previous definition, known as NSPs, which did not include lignins, RSs, fructans and other oligosaccharides. It was also proposed that the Association of Analytical Chemists methods should be used for the chemical determination of dietary fibre. For extracted natural and synthetic carbohydrates (e.g. FOS and GOS) to be defined as dietary fibre, there is a requirement for beneficial physiological effects to be demonstrated in the same way as for natural dietary fibres [15]. Following publication of the SACN report, the UK government changed dietary policy to be in line with its recommendations, which included increasing daily dietary fibre intakes for adults to 30 g [14]. All recommendations are shown below in table 3.

**Table 3.** Daily dietary reference values and intakes of dietary fibre for the average population groups within the UK using data from the National Diet and Nutrition Survey (NDNS) (2008−2019) [16]. DRV, dietary reference values.

population ages (years)	DRV (g d^−1^)	intake (g d^−1^)^a^
**children**		
2–5	15	11.5 ± 3.9
5–11	20	14.8 ± 4.2
11–16	25	15.6 ± 5.2
**adolescents**		
16–18	30 (about)	15.9 ± 6.1
**adults**		
19+	30	18.2 ± 6.9

^a^ Data represent mean ± s.d.

## 4. Population intake and dietary sources

**Figure 1.**
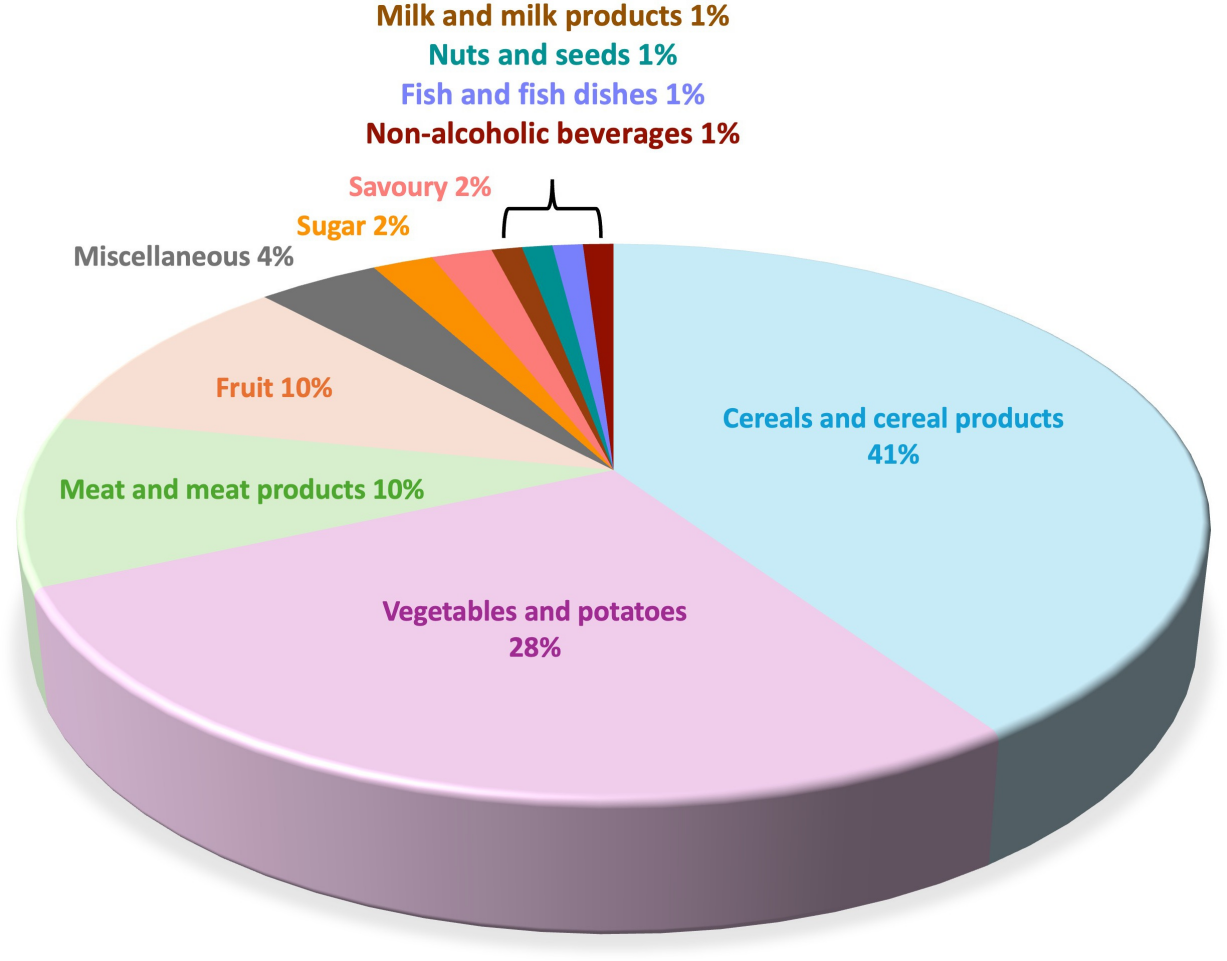
Summary of food groups contributing to average dietary fibre intake in the UK population (1.5−96 years) from the National Diet and Nutrition Survey (NDNS) (years 2008−2019) [16].

Despite the increase that was made almost a decade ago to the recommendations for dietary fibre intake, the mean population intake of all age groups in the UK is still well below dietary reference values (DRV; table 3). For example, daily dietary fibre intake in children is on average 3−9 g lower than recommended; but this disparity is greater in adults, with daily intakes of about 12−14 glower than the DRV. Hence, identifying foods that contribute to dietary fibre intake is fundamental to help guide relevant dietary strategies for the UK population. More specifically, data from the representative National Diet and Nutrition Survey (NDNS; cross-sectional rolling survey programme by the UK government [16]) captured dietary information, nutrient status and biomarkers of intake from a large sample size of 15 655 individuals (7656 children and 7999 adults) from populations aged 1.5 years and above, from 2008 to 2019 [16,17]. This was used to identify dietary fibre trends within the UK. Cereal-based products (41%), vegetables and potatoes (28%), meat-based products (10%) and fruits (10%) were found to contribute the majority of dietary fibre consumed by the UK population (figure 1).

In terms of specific food groups (table 4), rather than NDNS defined food categories (figure 1), the highest single contributors to fibre intake in the UK diet are pulses, of which the greatest one is baked beans. However, pulses only contribute a substantial amount of fibre for less than half of the UK population (39%). By contrast, staple foods such as bread are consumed by the majority of the population (i.e. 83% consume white and 54% high-fibre bread) and they are key contributors to dietary fibre intake (table 4). It is of note that some of the foods that contributed to dietary fibre intake are not typically recommended for consumption in large quantities (e.g. white bread, pizza and pies, where the fibre is mainly from cereal sources) according to the Eatwell guide [18].

**Table 4.** Top 12 commonly consumed foods that contribute to the average UK population dietary fibre intake (1.5–96 years) using data (mean intake and overall proportion of population consuming each food) from the National Diet and Nutrition Survey (NDNS) (years 2008−2019).

rank	foods	description	mean dietary fibre contribution	% of population consuming foods
1	pulses 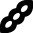	baked beans and dried peas, including chickpeas and lentils	7.6	39.1
2	high-fibre bread 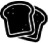	wholemeal, brown, granary, wheatgerm	7.4	54.1
3	breakfast cereals 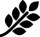	all cereals with a high fibre content	5.8	49.5
4	meat alternatives 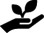	commercial meat alternative dishes (e.g. slices, pieces, mince, burgers, nuggets, pies, sausages, bacon strips) conatining plant-based alternatives (e.g. tofu, soya bean or mycoprotein) homemade meat alternative dishes (e.g. lentil lasagne)	5.6	4.6
5	potatoes 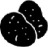	all dishes made from whole potato: homemade and manufactured	5.5	89.2
6	nutrition powders 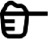	nutrient-packed vegetable and protein powders	4.9	1.6
7	pizza 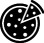	—	4.8	26.0
8	fruits 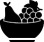	—	4.5	78.7
9	white breads 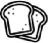	—	4.3	82.5
10	pasta 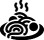	—	3.2	47.1
11	nuts and seeds 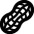	—	3.1	16.1
12	meat pies^a^ 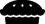	all pies/pastries made with meat	3.0	22.9

^a^ Fibre content of meat pies originates from plant ingredients (e.g. flour, any vegetables, etc.).

## 5. Strategies to increase dietary fibre intake

To increase daily dietary fibre intakes to recommended target levels, or at the very least ensure positive increases towards them, consideration needs to be given to different strategies for achieving this across a population. These include education, policy implementation, food labelling, (bio)fortification and supplementation, as described below.

### (a) Labelling

The likelihood of a strategy being successful is increased by consumers being able to easily engage with the dietary information and messaging on products at the point of purchase. Consumers need a system that enables them to quickly and clearly identify fibre content in the short time that they consider purchasing it, either in-store or online. Labelling needs to be obvious, clear and easy for consumers to understand. An example is the traffic light system approach that is currently used for energy, fat, sugar and salt, where green indicates low levels of dietary components compared with adult daily requirements, and red signals higher than recommended levels [19]. However, the current system would need to be modified to allow the importance of fibre content to be brought to the attention of consumers, enabling good (high) fibre content choices to be made and the success of any fibre campaign to be maximized. Currently, in Europe, a claim may only be made that a food is a ‘source of fibre’ when the product contains at least 3 g fibre/100 g fresh weight, whereas a claim that a food is ‘high fibre’ can be made only when the product contains at least 6 g fibre/100 g [20]. Approaches could include clear labelling of ingredients on the back of the food item (e.g. identification of the fibre components, such as inulin/guar gum, which are used as thickening or fat substitutes in some products), as well as the creation of a logo indicating higher-fibre foodsthat is displayed prominently on front of the pack, such as with the Danish Wholegrain Partnership (DWP).

Among the different strategies discussed here lies the question of health-by-stealth versus a public campaign focused on increasing knowledge of the importance of fibre and its health benefits, which enables consumers to make better product choices, e.g. wholemeal versus white bread. In the case of reducing sugar and salt intake in processed foods, numerous countries around the world have implemented a combination of strategies to reduce these ingredients within various products [21,22]. However, increasing an ingredient, such as fibre, in foods or drinks is different from decreasing ingredients that are known to have negative health effects (such as salt or trans-fat reduction), as some consumers distrust the addition of ‘unnecessary’ ingredients. To help increase consumption of foods with added ingredients (e.g. fibre), it is important to gain consumers’ trust by transparent labelling [23,24]. This is particularly the case for widely consumed staple products such as white bread in the UK.

### (b) Education

Consumers also need to have adequate knowledge of nutrition to help encourage healthy eating practices and understand relevant dietary recommendations [25]. More specifically, dietary fibre is associated with common misconceptions (e.g. fibre is brown, is it part of 5 a day? marketing linked with breakfast cereals, fibre leads to bloating, and so forth) [23]. Therefore, overcoming key knowledge gaps such as (i) health benefits beyond ‘digestive function’; (ii) challenges with identification of dietary fibre-rich foods; (iii) lack of awareness relating to daily dietary recommendations (30 g); and (iv) labelling that is difficult to interpret (e.g. small font size and back-of-pack), will be key in promoting dietary fibre intake at a population level [23,26–29]. This suggests that increasing awareness via targeted consumer-centric approaches is fundamental in driving such a shift.

Recently, Norton *et al*. [29] co-created dietary fibre-specific educational materials (in a factsheet and practical tips format) to promote intake based on the preferences of the target population (*n* = 150–170; adults aged 65 years and above). This approach was well received for all measured variables in terms of format, content, learning something new, modulating future intake and sharing materials with others (e.g. community, friends and family) [29]. However, future work needs to focus on quantifying dietary fibre intake post-engagement with educational materials. Information on dietary fibre intake is available from several organizations with details of the importance, recommendations, sources and tips for dietary inclusion (e.g. NHS [30], British Dietetic Association [31], British Nutrition Foundation [32]). Going forward, increased awareness and knowledge of available resources are essential for dietary change.

### (c) Reformulation, fortification and biofortification of foods

Given the alarming gap between actual and recommended intakes of dietary fibre (table 3) [33], long-term intervention at the educational level could be complemented by more immediate interventions to increase the fibre content of current diets, either by fortification or biofortification. Reformulation can be achieved by combining high- and low-fibre food sources, exemplified by the addition of a legume component with high endogenous fibre content to cereal flours. Legumes, being generally richer in several nutritional categories including protein, fibre and micronutrients, are attractive ingredients for a reformulation approach [34]. Fortification, on the other hand, involves the addition of fibre-rich fractions prepared from various plant sources to foods with low endogenous fibre contents. A wide range of such fractions is available commercially, but their incorporation into foods results in higher costs, which is an important concern in reaching lower-income groups and may also affect functionality, and hence product quality and consumer acceptability [35]. The extent to which added dietary fibre or fibre fractions have equivalent health benefits to endogenous fibre is also not known, as the latter form an intrinsic part of the food structure and may be more effective in limiting the rate of food breakdown and digestion in the GI tract. A more attractive strategy is, therefore, to increase the amounts of endogenous fibre present in food crops (biofortification) by selecting for natural variation in amount and composition. This should be achievable without significant effects on cost and processing quality (and hence acceptability), but requires long-term commitment bringing together crop geneticists, plant breeders and food processors [36].

### (d) Policy

Changing dietary intake to more healthy alternatives across the entire population is notoriously difficult to achieve [37]. Such changes are dependent on a range of factors including individual psychology, culture, socioeconomics and the political and cultural environment of the country in which the changes are being implemented. A government policy-driven approach, to which a population is receptive, is a key strategy for achieving positive dietary behaviour change *en masse*. Such policies, whether mandatory or voluntary, must collectively work with other sectors—notably the food industry in the case of fortification and reformulation—to enable change over a given timeframe and with realistic outcomes. Examples of this include the DWP, a joint public and private initiative that successfully increased daily cross-population wholegrain intake in Denmark from 36 g 10 MJ^−1^ in 2007 to 82 g 10 MJ^−1^ in 2019, the latter being 7 g 10 MJ^−1^ above the daily recommended target. Key to the success of the DWP was a focus on a familiar (high-fibre) staple food, rye bread and a strategy that included key food organizations such as the Danish Veterinary and Food Administration, public education on the importance of adequate whole grain consumption and its health benefits, and the development of a wholegrain logo that enabled consumers to easily identify wholegrain products. However, closer inspection of the data revealed minimal changes in certain sectors, including lower socio-economic groups who may benefit the most from these types of interventions. The approach has been considered for implementation in other countries, although such a strategy may not be as effective in countries with larger and more diverse populations such as the UK in which high-fibre breads (such as rye bread) are unfamiliar and poorly consumed [38]. While the national DWP programme proved successful in increasing wholegrain intake, alternative strategies also exist.

In the UK, current projects, funded by the Transforming the UK Food System (TUKFS; https://ukfoodsystems.ukri.org/) UKRI programme, are focused on providing healthy, sustainable diets for all with an aim of reducing diet inequities. They include projects that involve co-creation of more healthy foods, many of which are fibre-rich, that are acceptable to local disadvantaged communities. Such an example is the FoodSEqual project (for more information, see FoodSEqual [39]). Two further TUKFS projects are focused on increasing the fibre content of white bread, which is the greatest contributor to dietary fibre intake in the UK (figure 1). These projects are ‘Raising the Pulse’, which combines white wheat flour, a low-fibre ingredient, with a higher fibre ingredient such as nutrient-rich pulse flour obtained from faba beans [34] and Hi-Fi Bread that is focused on developing new cultivars of wheat via traditional breeding methods. The Hi-Fi bread project seeks to produce white flour with a higher fibre content, achieved by increasing the main fibre component of white flour, arabinoxylan, hence allowing fibre intake to be increased without changing dietary patterns [36]. Another example project, not within the TUKFS remit, is focused on increasing the fibre content of bread using a combination of cereals, brans, beans, peas and wheat germ [40]. These projects all focus on the improvement of white bread, a staple food that is consumed by over 83% of the UK population (table 4) and with which consumers are familiar from both cultural and historical perspectives.

## 6. Maintaining fibre supply in a changing world

Changes in agricultural practices in the UK, such as changes in land usage or the use of rotation systems, may be required to meet the growing demands for fibre-rich foods. An increased use of, for example, beans as a break crop, or under-sowing of beans, could be part of a strategy to increase bean production. This strategy also improves soil health, reduces disease pressure and may reduce nitrogen input requirements for the subsequent cereal crops. These approaches are becoming increasingly attractive since the production of oilseed rape—the dominant break crop used in rotation with wheat over the past few decades—has decreased owing to restrictions in pesticide use [41]. To increase the fibre content of bread, it will be necessary to ensure an adequate supply of raw materials (improved varieties of wheat and ‘new’ ingredients such as beans), which may require farmers to be financially incentivized. New baking and production methods should also be explored to ensure that these new or improved ingredients can be used on a commercial scale without prohibitive cost increases. In this sense, a whole food chain approach needs to be taken to ensure demands are met and the rollout of any strategic programme is effective.

All strategies require uninterrupted access to the required ingredients. In the case of bread, the UK generally produces around 85% of the wheat required, with the remaining 15% sourced internationally [42]. These sources vary geographically, but on the whole are readily available. However, the future reliance on international supplies needs to be questioned in a world of changing climate and protectionism against a backdrop of varying geopolitical events. The effects of these events on the resilience of food systems therefore need to be considered. Severe weather events can affect crop production within a country as well as imports to it, either by affecting crop production in the country of origin or transport of the crop (e.g. drought in the Panama Canal [43]). Geopolitical events, such as wars, can act in a similar way internationally, whereby crops become unavailable as a result of conflict within a country or through its inability to export the crop.

To ensure requirements are met, the design of our food system needs to move from a reactive perspective to one that is proactive, predictive and flexible. Weather model predictions exist that enable us to understand how extreme weather events can affect crop production both onshore and overseas [44]. Such models can also be used to mitigate disruptions in transport. By combining crop–climate and predictive supply chain models, we can begin to understand how our changing climate and approaches to mitigate fluctuations, such as stockpiling [45], can be used to support the uninterrupted supply of products (such as high-fibre foods) to consumers. Such models also provide scenario planning tools that can be used by components of the food chain, governments and policymakers to test how changes to the food system (e.g. increased land usage for onshore crop planting versus import storage capacity) can support the resilience of the supply of high-fibre foods to a country [46].

## 7. Summary and conclusions

Fibre is an important component of the diet, yet only 7.1% of the UK population meet current dietary recommendations [33]. Effective strategies are urgently required to increase intakes for health promotion and to prevent the current epidemic of non-communicable diseases, such as CVD, which is the greatest cause of mortality and morbidity worldwide. Such approaches should include, for example, better nutritional education and public health messaging, more informative and effective food labelling, food reformulation, fortification and biofortification, together with policy change. Success depends on the collective engagement of multiple stakeholders across the whole food system from consumers to farmers, food industry and government, as exemplified by the current UKRI-funded TUKFS initiative.

## Data Availability

Data described in the manuscript can be freely accessed from the UK Data services website [47]. The datasets used and analysed are available from the corresponding author on reasonable request.
